# Development of a Sensitive and Reliable Meso Scale Discovery-Based Electrochemiluminescence Immunoassay to Quantify TDP-43 in Human Biofluids

**DOI:** 10.3390/bios14120578

**Published:** 2024-11-28

**Authors:** Jiyan An, Lathika Gopalakrishnan, Vanessa Ortega, Justin Saul, Renu Kadali, Robert Bowser

**Affiliations:** 1 Department of Translational Neuroscience, Barrow Neurological Institute, Phoenix, AZ 85013, USA; jiyan.an@barrowneuro.org (J.A.); lathika.gopalakrishnan@barrowneuro.org (L.G.); vrortega04@gmail.com (V.O.); jpsaul@gmail.com (J.S.); renu.kadali@dignityhealth.org (R.K.); 2 Department of Neurology, Barrow Neurological Institute, Phoenix, AZ 85013, USA

**Keywords:** TDP-43, immunoassay, MSD, ALS, biomarker, neurodegenerative disease

## Abstract

Transactive response DNA-binding protein of 43 kDa (TDP-43) is a major component of pathological inclusions in various neurodegenerative disorders, including amyotrophic lateral sclerosis and frontotemporal lobar degeneration. The detection of TDP-43 in biofluids is crucial for the development of diagnostic and prognostic indicators of disease and therapeutic development for TDP-43-related proteinopathies. Despite its potential as a biomarker for numerous neurological disorders, the lack of a sensitive and reproducible TDP-43 assay hinders progress in TDP-43-based therapy development, underscoring the need for an effective and standardized method for accurate quantification. Addressing the limitations of sensitivity and reproducibility in existing assays, in this study, we developed and validated a highly sensitive electrochemiluminescence immunoassay on the Meso Scale Discovery platform. The assay demonstrated the detection of full-length TDP-43 in human biofluids with a limit of detection of 4pg/mL, a working range of 4–20,000 pg/mL, and a total assay time of 16 h. In this study, we developed and validated a sensitive immunoassay for the detection of full-length TDP-43 in human biofluids using the Meso Scale Discovery platform. We used this immunoassay to quantify TDP-43 levels in the plasma and serum of healthy controls and ALS patients. Our results indicate a reduction in full-length TDP-43 in the blood of ALS patients compared to healthy controls.

## 1. Introduction

Neurodegenerative disorders pose a significant and escalating challenge to global healthcare, with a growing impact on quality of life for millions worldwide. Among these disorders, amyotrophic lateral sclerosis (ALS) is a fatal motor neuron disease characterized by progressive muscle weakness, paralysis, and ultimately, respiratory failure [1]. Despite extensive research efforts, the precise etiology of ALS remains elusive, necessitating a deeper understanding of the molecular mechanisms driving the neurodegenerative processes underlying this debilitating condition.

In recent years, attention has turned towards the role of TAR DNA-binding protein 43 (TDP-43), a nuclear ribonucleoprotein, in the pathogenesis of ALS. Originally identified for its involvement in RNA processing, TDP-43 has emerged as a key player in neurodegeneration, with its aggregation and mis-localization being recognized as prominent pathological hallmarks in ALS and other related disorders such as frontotemporal lobar degeneration (FTLD), limbic-predominant age-related TDP-43 encephalopathy (LATE) [2], and Alzheimer’s disease [3,4,5]. TDP-43 likely functions as a key mechanistic contributor in both sporadic and most familial forms of ALS and is a neuropathologic feature in over 95% of all ALS [6].

TDP-43 is ubiquitously expressed in the central nervous system and plays a crucial role in RNA metabolism, including transcription, splicing, and transport [7]. Under normal physiological conditions, TDP-43 is predominantly localized in the nucleus, contributing to the maintenance of cellular homeostasis [8]. However, in ALS and a spectrum of related neurodegenerative disorders collectively known as TDP-43 proteinopathies, this protein undergoes aberrant modifications, leading to its mislocalization from the nucleus and generation of cytoplasmic inclusions, leading to neuronal dysfunction and degeneration [9]. The cytoplasmic aggregation of TDP-43 has been observed in the affected regions of the central nervous system in ALS patients, including the spinal cord and motor cortex. TDP-43 inclusions in ALS and FTLD contain an accumulation of native protein but also hyperphosphorylated and truncated forms of the TDP-43 protein [9].

The correlation between the presence of these TDP-43 aggregates and the severity of clinical parameters of disease underscores the significance of understanding the mechanisms governing TDP-43 pathology. Currently, the detection of these pathologic aggregates is limited to postmortem neuronal tissues. Moreover, emerging evidence suggests that TDP-43 mislocalization may precede the onset of clinical symptoms, pointing towards its potential role as an early contributor of disease [10]. The cytoplasmic accumulation of TDP-43 suggests that TDP-43 may ultimately be released into the extracellular space via cell degeneration, the exosome, or autophagosome release from cells [11,12]. Reliable detection of TDP-43 in biofluids could serve as a biomarker for TDP-43 proteinopathies, as well as for the development of therapeutic strategies that target TDP-43 function. While several immunoassays have been developed for TDP-43 detection, including enzyme-linked immunosorbent assay (ELISA) and Western blot, the current assays have limitations in terms of sensitivity and reproducibility [13,14]. Here, we describe a Meso Scale Discovery (MSD)-based immunoassay for the detection of TDP-43 in blood using an antibody pair that detects the full-length TDP-43 protein and potential fragments containing the N-terminal region of the protein.

MSD is a highly sensitive and versatile immunoassay platform that leverages electrochemiluminescence (ECL) technology for the detection and quantification of proteins, peptides, and other biomolecules [15]. The detection of the target protein involves two key antibodies: (1) a capture antibody, which specifically binds to the target protein (in this case, TDP-43), and (2) a detection antibody that binds to the target protein, forming a sandwich that is then recognized by an anti-species-specific antibody to the detection antibody labeled with an electrochemiluminescent tag, such as the SULFO-TAG (Supplementary Figure S1A). The ECL signal is generated through a sequence of electrochemical reactions facilitated by electrodes embedded in the assay plate. When an electric current is applied in the presence of a co-reactant solution containing tripropylamine (TPrA), the Ruthenium-based SULFO-tag undergoes oxidation, causing a cascade of reactions that trigger the labeled antibodies to produce a luminescent signal. The emitted light is detected and quantified, correlating with the amount of analyte captured in the immunoassay. The signal intensity of this light is directly proportional to the concentration of the target analyte, enabling a quantitative measurement of the analyte in the sample (Supplementary Figure S1B). MSD assays have been successfully applied to detect and quantify various proteins associated with neurodegenerative diseases. For example, in Alzheimer’s disease (AD) research, MSD has been used to measure the levels of amyloid-beta (Aβ) and tau proteins in cerebrospinal fluid (CSF), both of which are key biomarkers for the diagnosis and progression of AD [16,17]. In Huntington’s disease (HD), MSD has been utilized to quantify huntingtin (HTT) protein levels, helping to track disease progression and evaluate therapeutic interventions [18]. Similarly, our use of the MSD platform in the quantification of full-length TDP-43 in biofluids will be a valuable tool to measure levels in many neurodegenerative diseases and evaluate alterations due to pathophysiologic changes in TDP-43 and responses to treatments that target TDP-43 in ALS as well as other TDP-43 proteinopathies.

## 2. Materials and Methods

### 2.1. Materials

**Figure 1:**
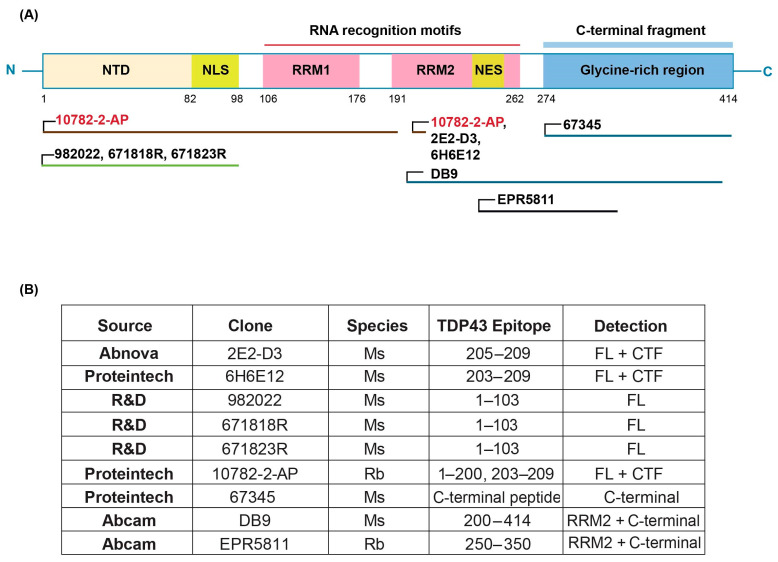
Schematic representation of the MSD-based TDP-43 immunoassay. (**A**) The structure of the TDP-43 protein and localization of epitopes against different antibodies screened in this study. All the monoclonal antibodies are marked in black and the polyclonal antibodies in red. (**B**) Source and description of each commercial antibody used in this study.

Purified recombinant transactive response DNA-binding protein of 43 kDa (TDP-43) was purchased from OriGene (NM_007375, Cat#TP710010; Rockville, MD, USA) and used as the assay calibrant. Antibodies and their source are shown in Figure 1. TDP-43 Rabbit Polyclonal antibody (Proteintech, Catalog #10782-2-AP; Rosemont, IL, USA) and Human TDP-43/TARDBP Mouse monoclonal antibody, clone 671818R (R&D Systems, Catalog #MAB77782-100l; Minneapolis, MN, USA) were found to be the optimal capture and detection antibodies, respectively. For immunodepletion experiments, we used 7 different TDP-43 antibodies including TARDBP monoclonal antibody (M01), clone 2E2-D3 (Abnova Catalog # H00023435-M01; Taipei City, Taiwan); TDP-43 (human specific) monoclonal antibody, clone 6H6E12 (Proteintech Catalog # 60019-2-Ig); Human/Mouse/Rat TDP-43/TARDBP Antibody, clone 982022 (R&D Systems Catalog #MAB77781); Human TDP-43/TARDBP Mouse monoclonal antibody, clone 671818R (R&D Systems, Catalog #MAB77782-100); Mouse monoclonal anti-TDP43 Antibody (Biogen Clone # L95A42.1; Cambridge, MA, USA); TDP-43 (C-terminal) Monoclonal antibody (Proteintech Catalog # 67345-1-Ig); and Anti-TDP43 antibody DB9 (Abcam Catalog # ab254166; Cambridge, UK).

The calibrant and sample diluent, Iron Horse Assay Diluent (IHAD), was purchased from nVector (Catalog #AD01-10, Phoenix, AZ, USA). SULFO-TAG labeled anti-mouse antibody (Catalog #R32AC-1), Read buffer A (Catalog #R92TC), and MSD SECTOR Plates (Catalog #L15XA) were obtained from Meso Scale Discovery (Rockville, MD, USA).

### 2.2. Sample Collection and Preparation

Plasma and serum samples from ALS and healthy control participants after IRB approved consent were obtained from the NEALS Biorepository (https://neals.org/als-researchers/neals-sample-repository (accessed on 10 January 2024), Boston, MA, USA) and St. Joseph’s Hospital and Medical Center Biobank (Phoenix, AZ, USA) using standardized protocols. Briefly, blood was collected in K2EDTA tubes, inverted multiple times, and centrifuged at 1500× *g* for 10 min. The plasma was carefully collected, aliquoted, and stored at −80 °C until analysis. The serum was isolated by collecting blood in red-top tubes lacking anticoagulant, allowed to clot at room temperature for one hour, and centrifuged at 1100× *g* for 15 min. The serum was collected, aliquoted, and stored at −80 °C for subsequent analysis.

### 2.3. Meso Scale Discovery (MSD) Assay Conditions

The recombinant full-length TDP-43 calibrator purchased from OriGene was diluted with IHAD to a final concentration of 1ug/mL as a standard stock, aliquoted at 20 µL per tube, and stored at −80 °C. For each standard curve, 980 µL of 20% IHAD was added directly to the standard stock tube and mixed well. A series of 4-fold dilutions with 20% IHAD was performed to generate a standard range from 20,000 pg/mL to 4.88 pg/mL. Standard 8 was 20% IHAD only as a blank. MSD standard sector single-spot plates were coated with 40 µL of 1.5 µg/mL Rb poly anti-human TDP-43 as the capture antibody (ProteinTech, Cat#10782-2-AP) in PBS overnight at 4 °C. Plates were washed with 250 µL of wash buffer (PBST-0.1% Tween 20 in 1X PBS) 4 times then blocked with 1%BSA in 1% Casein/TBS (Bio-Rad, Cat#1610782; Hercules, CA, USA) at 37 °C for 2 h with shaking at 700 rpm using a plate shaker. The plasma or serum samples were diluted 4-fold in 20% IHAD. After the plates were washed 4 times in PBST, 40 µL of standards or diluted samples were added to an MSD microplate. The plates were incubated at 4 °C overnight with shaking at 750 rpm using Heidolph Titramax 1000 (Schwabach, GER). After 4 washes with 250 µL of PBST, 40 µL of 1.0 µg/mL mouse monoclonal anti-human TDP-43 as the detection antibody (R&D Systems, Cat#MAB77782-100) in Casein/TBS was added to each well and incubated with shaking at 37 °C for 2 h. After plate washes (250 µL of PBST 4 times), 40 µL of SULFO-tagged anti-mouse antibody (1.0 µg/mL MSD, Cat#R32AC-1) in Casein/TBS was added to each well and incubated with shaking at 37 °C for 2 h. After final plate washes (250 µL of PBST 4 times), 150 µL of Read Buffer A (MSD, Cat#R92TG) was added to each well to initiate the electrochemical reaction and immediately read on an MSD MESO QuickPlex SQ120. The standard curve was generated using serial dilutions of recombinant TDP-43, and the signal intensities from the samples were interpolated onto the standard curve, converting the intensity value into corresponding concentration values of TDP-43. The detailed assay protocol is provided in Appendix A.

### 2.4. MSD Assay Performance and Reproducibility

Validation studies were performed to evaluate the reproducibility, precision, dilution linearity, and specificity of the final assay.

#### 2.4.1. Reproducibility and Precision

To determine the reproducibility and precision of the assay, we evaluated the precision (intra- and inter-assay variability) of the assay. Intra-assay variability was assessed by repeating samples on the same plate to test within run variations of the assay. Inter-assay variability was determined by measuring the mean precision (coefficient of variability) between assays performed using the same conditions on different days.

#### 2.4.2. Dilution Linearity

The linearity of the assay across dilution ranges was assessed to ensure the accurate quantification of TDP-43 levels. The plasma and the serum samples were serially diluted 2-fold 6 times, yielding dilutions ranging from 2-, 4-, 8-, 16-, 32-, and 64-fold. The measured final concentrations were determined and then compared with the calculated concentrations, considering the relevant dilution factor. 

#### 2.4.3. Spike-In Recovery

Experiments were conducted to evaluate the assay accuracy and matrix effects under controlled conditions. A defined concentration (5 ng/mL) of human recombinant TDP-43 protein was added to plasma and serum samples in different dilutions ranging from 2-fold to 64-fold. Samples with no protein spiked in served to define the endogenous amount of TDP-43 protein in the sample. The TDP-43 protein concentration was determined in all samples to evaluate any matrix effect on the total TDP-43 protein measured in all samples.

#### 2.4.4. Specificity Test

To demonstrate specificity of the assay for TDP-43 in a human biofluid, we removed TDP-43 from human biofluid samples by immunoprecipitation pull-down of endogenous TDP-43 using the mouse monoclonal antibodies 671818R or 982022 (Section 2.1). Human biofluid samples were pooled from healthy controls, and immunoprecipitation was performed using Dynabeads (ThermoFisher Scientific, Waltham, MA, USA) with 4 µg of each of the TDP-43 antibodies. The samples were incubated with the antibody overnight at 4 °C with high agitation (workflow depicted in Supplementary Figure S2). Following incubation, the antibody–protein complexes were pulled down using protein G magnetic beads. The supernatant, containing the immunodepleted samples, was collected for further analysis. The immunodepleted samples were then analyzed using the MSD assay to quantify full-length TDP-43 and the results were compared to those obtained from non-depleted control samples. As controls, we used a non-specific IgG antibody, and the MSD assay was performed on both the IgG/TDP-43 antibody as well as the pooled sample that was not depleted.

### 2.5. TDP-43 Measures in Clinical Samples

The final assay performance was tested using a set of samples from ALS subjects and healthy controls, obtained from the NEALS Biorepository as well as St. Joseph’s Hospital and Medical Center Biorepository. Full-length TDP-43 was measured in 100 plasma and 100 serum samples (unmatched) from ALS and healthy control subjects constituting a total of 400 biofluid samples. A quality control sample was included in all the plates to evaluate the inter-plate variations and consistency in the results.

### 2.6. Figures and Tables

The graphs were generated using GraphPad Prism version 10.1.0 GraphPad Software, Boston, Massachusetts USA. Figures were generated using Adobe Illustrator (version 27.3.1; San Jose, CA, USA) and BioRender.com (Premium) (Toronto, ON, CA).

## 3. Results

### 3.1. Antibody Selection for Assay Development

We used the Meso Scale Discovery (MSD) platform to develop the current full-length TDP-43 immunoassay, which utilizes electrochemiluminescence to detect proteins with high sensitivity and specificity (Supplementary Figure S1A). The first step was to identify the best antibody pair to use for capture and detection in the assay. Figure 1A,B) specifies the source and location of the antibody epitopes used to develop the current assay. We first performed a dot blot analysis using both the purified recombinant TDP-43 protein and human frontal cortex sarkosyl tissue extracts to confirm that the antibodies listed in Figure 1B detected the recombinant TDP-43 protein and the protein in a complex tissue extract. All antibodies detected both the recombinant and TDP-43 protein in the tissue extracts. We next performed all possible pairwise combinations of antibodies to determine capture and detection antibody pairs with maximal signal-to-noise ratio using the purified recombinant human TDP-43 protein spiked into tris buffered saline (TBS) containing 1% Casein or human CSF. Several antibody pairs detected the recombinant TDP-43 protein when spiked into buffer or CSF, and these pairs were then tested for their ability to detect the TDP-43 protein in a human frontal cortex sarkosyl tissue extract. Specifically, the configuration using 10782-2-AP as the capture antibody and 671818R as the detection antibody exhibited the highest S/N ratio in plasma and serum. This ultimately guided the selection of 10782-2-AP as capture and 671818R as detection antibodies for our assay conditions. Based on the known epitopes, this antibody pair should detect the full-length TDP-43 protein and any N-terminal fragments of TDP-43.

### 3.2. Optimization of Assay Parameters

Several parameters were optimized during the development of this electrochemiluminescence assay to enhance its performance. Table 1 highlights the MSD assay parameters optimized with the selected antibody pair. We tested several candidate sample diluents to identify one that maintained the stability of the antibodies and TDP-43 protein in biofluids to enable optimal assay performance. The selected diluent, Iron Horse Assay Diluent (IHAD), facilitated maximal binding and specificity of the antibodies in the assay (Supplementary Figure S3B). Various blocking reagents were evaluated to identify the one most effective for minimizing the background signal and enhancing the signal. Simultaneously, we evaluated the effects of temperature and shaking of the plate for each blocking reagent. While different blocking buffers showed varying results, the incubation time and temperature yielded similar outcomes. After evaluating different conditions, we selected 1% BSA in Casein/TBS as the blocking reagent and performed a 2-h incubation at 37 °C with shaking. This incubation time and temperature was opted for since all the tested conditions yielded similar results, and the 2-h incubation provided the shortest effective blocking time (Supplementary Figure S3C,D). Next, we tested various sample dilutions to ensure accurate quantification of TDP-43 levels without interference from the complex biofluid matrix. In Supplementary Figure S3E,F, we show how the assay performed across different dilution factors of plasma and serum samples, ranging from 2-fold to 256-fold for plasma and from 2-fold to 64-fold for serum. While TDP-43 detection remained stable across the dilution range from 4-fold to 16-fold, we opted to use a 4-fold dilution in the assay. This decision was made to account for the heterogeneity in TDP-43 concentrations across samples, particularly in cases with very low TDP-43 levels. By selecting a lower dilution factor, we ensured the assay maintained its sensitivity for detecting low analyte concentrations while preserving robustness.

We next determined the antibody concentrations optimal for the assay. We varied the capture antibody concentrations used to coat plates from 0.5 µg/mL to 1.5 µg/mL to determine the levels that provided the best balance between capture efficiency and signal intensity. We found that 1.5 µg/mL of the capture antibody yielded the maximum detection sensitivity of and dynamic range for measuring TDP-43 protein concentration in human biofluids (Supplementary Figure S4A). The concentration of the detection antibody was also varied between 0.5 µg/mL and 1.5 µg/mL to optimize assay detection and to achieve the ideal balance between sensitivity and specificity. We found that the optimal detection antibody concentration was 0.5 µg/mL, providing the highest signal-to-noise ratio and dynamic range and assuring the accurate quantification of TDP-43 levels in human biofluids (Supplementary Figure S4B). The concentration of the SULFO-tag anti-mouse antibody was optimized to ensure efficient electrochemical signal generation. A concentration of 1.0 µg/mL SULFO-tag labeled anti-mouse in 1X TBS with 1% Casein provided optimal sensitivity and accuracy of the immunoassay (Supplementary Figure S4C).

### 3.3. Precision, Sensitivity, and Specificity

**Figure 2:**
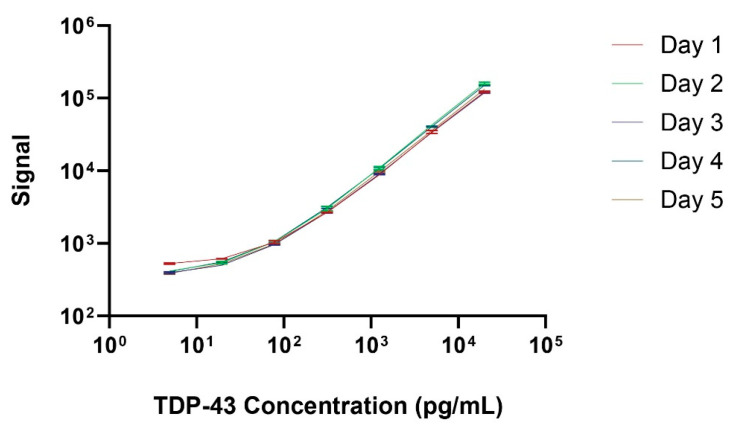
Representative TDP-43 standard curves generated on five consecutive days. The MSD signal is shown in the Y-axis and the TDP-43 concentration in the X-axis. Error bars representing standard deviation (SDs) are included for each standard curve from replicate samples at each protein concentration.

**Figure 3:**
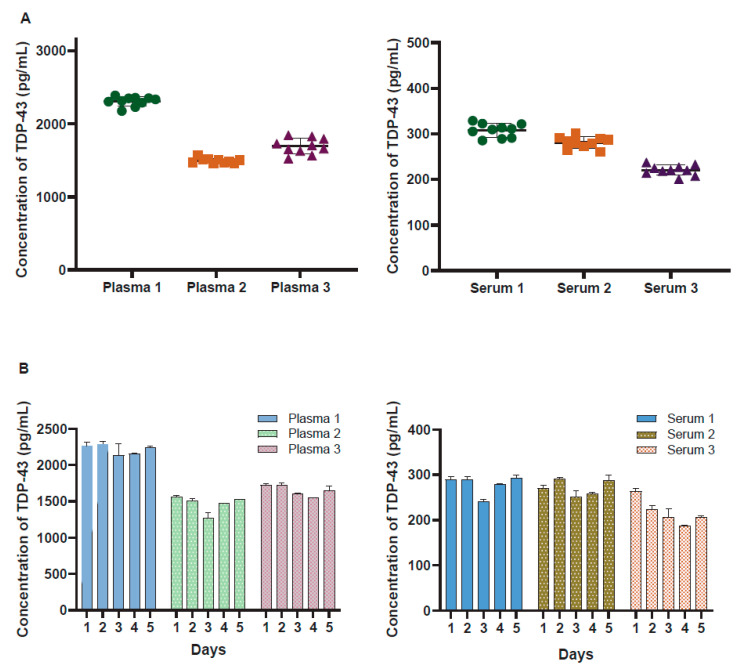
Assessment of precision and sensitivity of the TDP-43 assay. (**A**) Intra-assay TDP-43 concentration values for 10 replicates of 3 separate plasma (left) and serum (right) samples. The intra-assay coefficient of variation (CV) ranged from 2.7% to 6.3% in plasma and from 4.9% to 5.0% in serum, indicating minimal variability within the same day. (**B**) Inter-assay variability observed in the assay across multiple replicates of the assay conducted on different days for 3 separate plasma and serum samples. Each bar represents the average of replicate wells for each sample, with error bars representing +/− SD for each sample.

Reproducibility and repeatability were assessed via ten replicates on a single plate for intra-assay variability and repeating the same assay on five consecutive days to evaluate inter-assay variability. The coefficient of variation (CV) was calculated for both the intra-assay (within the same day) and inter-assay (across different days) analyses. Figure 2 illustrates the standard curve over 5 separate days, with a lower limit of quantification of 4.0 pg/mL and an upper limit of quantification of 20,000 pg/mL. Figure 3A displays the intra-assay TDP-43 concentration values for 10 replicates of three separate plasma (left) or serum (right) samples. The intra-assay CV ranged from 2.7% to 6.3% in plasma and from 4.5% to 5% in serum, indicating minimal variability within the same day. The inter-assay results shown in Figure 3B represent a dilution series of TDP-43 measured on 5 consecutive days. The CV ranged from 3.6% to 4.8% in plasma and from 5.7% to 8.8% in serum, reflecting consistent performance across different days. These results underscore the precision and reliability of the assay for quantifying TDP-43 levels in biofluids.

**Figure 4:**
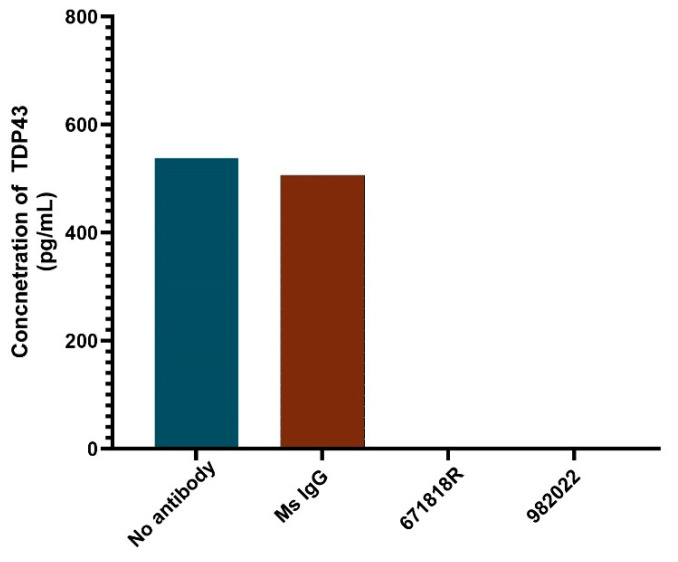
Immunodepletion of TDP-43 demonstrates immunoassay specificity. Plasma was incubated with no antibody (blue bar), control IgG (red bar), anti-TDP43 antibody 671818R, or anti-TDP43 antibody 982022, and immunodepletion was performed as described in the Materials and Methods. Immunodepleted samples were analyzed for TDP-43 protein levels, and the average protein concentration from replicate samples is displayed. Samples depleted with the anti-TDP43 antibody were devoid of the TDP-43 signal.

To assess the specificity of the assay, we depleted endogenous TDP-43 from a pooled plasma sample from healthy controls by immunoprecipitation using two different TDP-43 antibodies or an IgG control antibody that does not recognize TDP-43. The starting sample and three immunodepleted samples were subsequently used to measure TDP-43 levels with our immunoassay. The starting sample exhibited a TDP-43 protein concentration of approximately 550 pg/mL (Figure 4). The non-specific IgG control failed to remove TDP-43 and exhibited a TDP-43 concentration similar to the starting sample, whereas those samples incubated with either of the TDP-43-specific antibodies for immunodepletion exhibited a complete loss of the TDP-43 signal (Figure 4). These results confirm that the signal detected with our immunoassay is dependent upon the presence of TDP-43 and is not cross-reactive with other proteins within the biofluid.

### 3.4. Freeze–Thaw Stability

**Figure 5:**
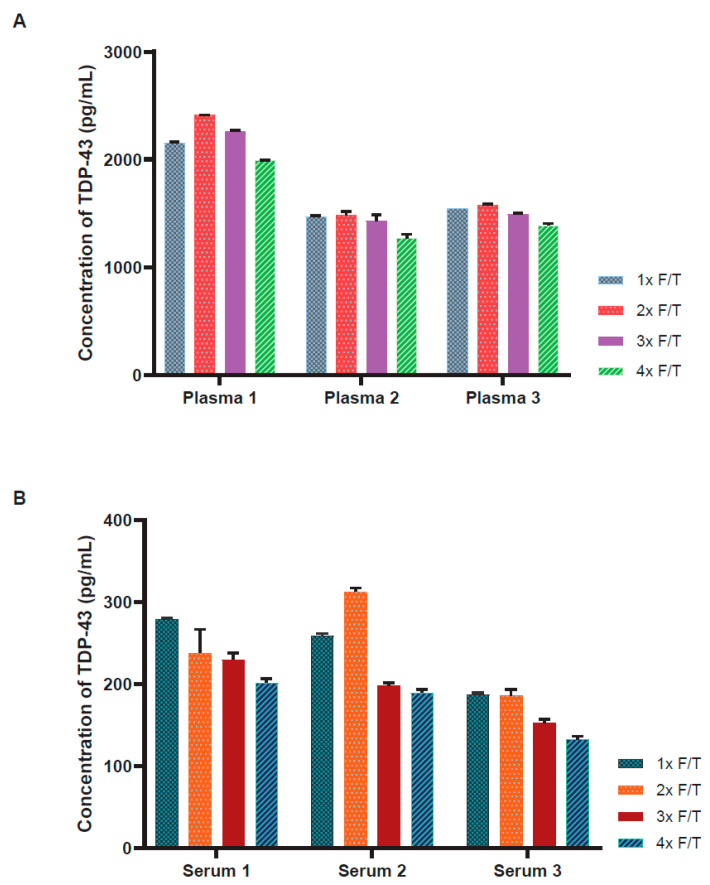
Stability of endogenous TDP-43 in plasma and serum across freeze–thaw cycles. The bar plots represent the freeze–thaw stability of TDP43 across four freeze–thaw cycles of plasma (**A**) and serum (**B**). The results show minimal impact on assay performance and TDP-43 protein concentrations across the first two or three cycles. A modest reduction in TDP-43 concentration was noted in the fourth freeze–thaw cycle for plasma and the third or fourth freeze–thaw cycle for serum. The bars represent the average of replicate samples for each sample, with error bars representing +/− SD for each sample.

The stability of endogenous TDP-43 to numerous freeze–thaw events was assessed next. To evaluate the impact of freeze–thaw cycles on TDP-43 stability and assay performance, multiple freeze–thaw cycles were performed on plasma and serum from three subjects stored at −80 °C. The freeze–thaw results demonstrated a negligible impact on assay performance and observed TDP-43 protein concentration after the first few freeze–thaw cycles, though a modest concentration reduction was observed in the fourth freeze–thaw cycle for plasma (Figure 5A) and either the third or fourth freeze–thaw cycle for serum (Figure 5B).

### 3.5. Parallelism and Spike-In Recovery

**Figure 6:**
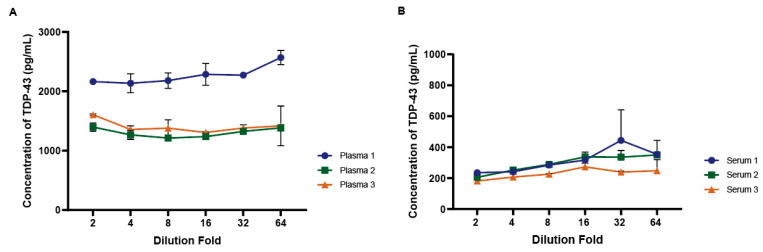
Dilution linearity of the plasma and serum samples using the TDP-43 assay. Panel (**A**) and (**B**) illustrate the dilution linearity assessment in plasma and serum, respectively. A linear relationship between dilution factors and TDP-43 concentration was observed both in plasma and serum, with the assay maintaining a stable measurement of TDP-43 even at dilutions up to 64-fold. Error bars (+/− SD) are shown for each sample.

Parallelism was assessed to ensure the assay’s accuracy across a range of sample concentrations and minimal impact of the matrix at the specified dilutions. Parallelism addresses the relative accuracy of the assay by assessing the effects of dilution on the quantitation of TDP-43 in a biologic matrix. As shown in Figure 6, the dilution linearity or parallelism assessment revealed a linear relationship up to a 64-fold dilution across multiple plasma (A) or serum (B) samples. This indicates the assay’s ability to accurately quantify TDP-43 levels over a broad range of plasma or serum dilutions with a limited impact on overall concentration values.

**Figure 7:**
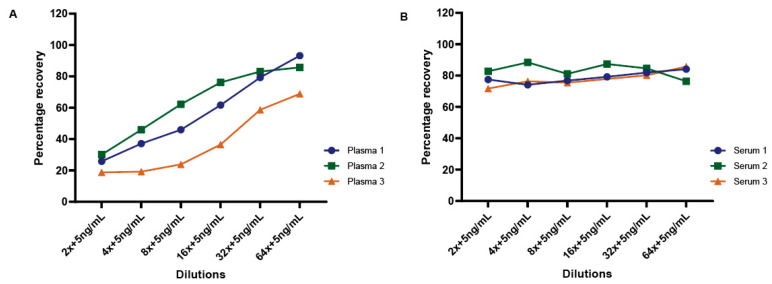
Spike-in recovery of TDP-43 in human plasma and serum. A fixed concentration (5ng/mL) of human recombinant TDP-43 was spiked into plasma and serum samples at various dilutions (2-fold to 64-fold). The graph illustrates the recovery rates of TDP-43 both in plasma (**A**) and serum (**B**).

To further evaluate matrix effects on measuring TDP-43 protein concentrations in human biofluids, we performed spike-in recovery tests in both plasma and serum samples. A defined concentration (5.0 ng/mL) of the purified human recombinant TDP-43 protein was added to plasma and serum samples at different dilutions ranging from 2-fold to 64-fold. Samples with no spiked-in protein served to define the endogenous amount of TDP-43 protein in the sample. As shown in Figure 7, the results revealed a variable matrix effect trend across all plasma samples, with recovery rates increasing with plasma dilution (Figure 7A). However, the serum samples exhibited recovery rates of approximately 80–90% at all dilutions (Figure 7B).

### 3.6. Quantification of TDP-43 in Blood Samples of ALS Patients and Healthy Controls

**Figure 8:**
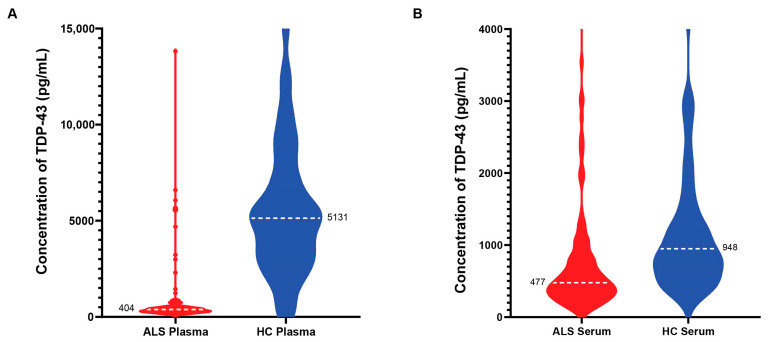
The quantification of TDP43 human plasma and serum samples. The violin plots represent the TDP-43 protein levels quantified in ALS (n = 101) and control (n = 115) plasma (**A**) and serum samples (**B**), with mean levels represented as the dotted white line.

We used our MSD assay to quantitate the full-length TDP-43 in plasma and serum (unmatched) from 101 ALS and 115 age-matched healthy control subjects. The limit of quantitation (LLOQ) was 4.0 pg/mL in serum and plasma with a dynamic range of 4–20,000 pg/mL. All plasma and serum samples fell into the working range of this assay. We observed a decrease in full-length TDP-43 in the plasma of ALS patients when compared to healthy control samples (Figure 8A). The ALS plasma group displayed a mean of 404 pg/mL (95% CI of the mean between 330 and 425 pg/mL) versus the healthy control mean of 5131 pg/mL (95% CI of the mean between 4379 and 5512 pg/mL), with *p* = 0.001 using an unpaired *t*-test. We also detected significantly lower levels of TDP-43 in the serum of ALS patients when compared to age-matched healthy controls (Figure 8B). The mean TDP-43 concentration in the ALS serum group was 477 pg/mL (95% CI of the mean between 430 and 573 pg/mL) versus that of a 948 pg/mL mean in the healthy controls (95% CI of the mean between 765 and 1108 pg/mL), with *p* = 0.001 using an unpaired *t*-test.

## 4. Discussion

The present study addresses the growing body of evidence supporting the need for improved immunoassays to measure TDP-43 in ALS and related disorders. Our study defines steps and conditions to create a reliable and reproducible immunoassay for quantifying full-length TDP-43 in human biofluids. The final capture and detection antibodies as well as all the reagents are commercially available. The assay protocol as well as the capture 10782-2-AP polyclonal antibody are freely available to the research community via Target ALS (https://www.targetals.org/resource/antibody-core/, accessed on 11 October 2024).

Additionally, the potential application of our novel assay in the diagnostic work-up for dementia disorders, including the detection of brain TDP-43 deposition, holds promise for improving clinical diagnosis and patient management. Further validation studies in larger patient cohorts are warranted to confirm the robustness and clinical utility of our novel TDP-43 assay in neurodegenerative disorder diagnosis and research. However, it is crucial to acknowledge that while our results indicate a correlation between reduced full-length TDP-43 levels and ALS, further investigations are necessary to unravel the underlying biology of this reduction.

### 4.1. Antibody Screening

We screened multiple commercially available TDP-43 antibodies during immunoassay development, as antibody specificity and affinity greatly influence assay performance. In our study, we rigorously evaluated a panel of antibodies targeting different epitopes of TDP-43 (Figure 1B) to identify antibodies that detected TDP-43 in tissue extracts, dot blots of human biofluids, and pure TDP-43 protein spiked into human biofluids to define an optimal antibody pair for sandwich immunoassay configuration. Despite extensive screening efforts, challenges such as antibody cross-reactivity or poor specificity were noted for some antibodies. To mitigate these issues, we employed stringent validation criteria and confirmed antibody specificity, ensuring the reliability of antibodies used in our final immunoassay. We tested multiple wash conditions, assay diluents, and incubation times and temperatures to maximize the assay signal and minimize the background (Table 1). This screening process enabled us to develop an assay with high sensitivity in human plasma and serum samples, as evidenced by the lower limits of detection (LODs) and quantification (LLOQs) achieved.

The nature of the TDP-43 protein presents additional challenges in its detection and quantification. TDP-43 is known to undergo fragmentation, aggregation, and formation of insoluble protein aggregates observed in neurodegenerative disorders, including ALS and FTLD [19,20]. The detection of soluble and insoluble forms of TDP-43 poses challenges for immunoassay development, though the detection of pathologic species of TDP-43 is crucial for generating biomarkers of disease and monitoring the impact of TDP-43 based therapies in clinical trials [6,21,22]. Based on the epitopes detected by each antibody selected in our screening procedure, our immunoassay will detect full-length or N-terminal fragments of TDP-43. Since N-terminal fragments of TDP-43 have not been observed in human tissue extracts or biofluids, we propose that our assay detects full-length TDP-43.

Experiments examining the matrix effects of plasma and serum demonstrated that dilutions of either serum or plasma samples provided a linear relationship of measured TDP-43 concentration, though higher dilutions of some samples generated increased TDP-43 protein concentrations (Figure 6). This result suggests that a minimal matrix effect may occur in some human plasma or serum samples. However, our spike-in recovery experiments suggest that plasma has a more significant matrix effect and that spike-in recovery in serum was comparable across a series of sample dilutions (Figure 7). A reduced recovery was observed in the lower dilutions of plasma when compared to the higher dilutions. Overall, we observed that we could specifically and accurately detect TDP-43 in either plasma or serum, but plasma samples may contain either other proteins, cell types, or lipids that impede the detection of exogenously added TDP-43.

### 4.2. Decrease in TDP-43 in ALS Plasma and Serum

Contrary to previous reports suggesting elevated TDP-43 levels in ALS blood samples compared to healthy controls [23], our results revealed a significant decrease in full-length TDP-43 in ALS patients compared to healthy controls (Figure 8). Similarly, another study also suggested lower levels of TDP-43 in the plasma of ALS patients from an Indian population compared to controls by immunoassay [24]. A recent study that validated a TDP-43 immunoassay for detecting C-terminal fragments of TDP-43 reported increased TDP-43 C-terminal fragments in human plasma from ALS patients [23]. These authors observed between 50 and 100 pg/mL of C-terminal TDP-43 in plasma samples, while we typically detected low ng/mL levels of full-length TDP-43 in plasma. Additionally, reductions in total TDP-43 levels have been reported in the lumbar cerebrospinal fluid (CSF) of FTD-TDP patients. In this study, lower CSF TDP-43 levels were correlated to levels of TDP-43 pathology in tissue, suggesting that total TDP-43 could serve as a biomarker for predicting TDP-43 neuropathology in tissue [25]. Moreover, this study proposes that reduced CSF TDP-43 levels in FTD patients was associated with the presence of the C9ORF72 repeat expansion. Overall, these results suggest that our observed reduction of full-length TDP-43 in the blood of ALS patients may correlate with an increase in C-terminal fragments in the plasma of ALS patients or is possibly due to the distinct stage of disease progression represented by the samples used in this study.

The observed reduction in full-length TDP-43 levels in ALS versus healthy control blood samples raises intriguing questions regarding the underlying mechanisms. The decrease in full-length TDP-43 levels may reflect dysregulation in the processing or turnover of this essential RNA-binding protein, which is known to play a crucial role in RNA metabolism and maintaining cellular homeostasis. TDP-43 abnormalities have been extensively linked to ALS, and our findings further strengthen the association between ALS and TDP-43 concentrations in serum and plasma. It is well established that TDP-43 undergoes various post-translational modifications, including proteolytic cleavage, which can lead to the generation of C-terminal fragments and degradation of the N-terminus [8,26]. While our results suggest a correlation between reduced full-length TDP-43 levels in the blood and ALS, additional research is necessary to define the mechanisms leading to this reduction and any correlation to TDP-43 pathology with the brain and spinal cord. It is possible that TDP-43 levels in the blood may differ at different disease stages or vary within different blood cell types or biofluid matrices. A prior study suggested that higher levels of TDP-43 occur in platelets of ALS patients [27]. Further studies are required to determine if TDP-43 within the plasma or serum is contained within exosomes or extracellular RNPs or is localized within a particular cellular component of the plasma or serum, such as platelets [21,28].

Future studies should focus on generating additional immunoassays that specifically quantitate post-translational modifications of TDP-43. Additionally, exploring the correlations between full-length, C-terminal fragments and post-translational modifications of TDP-43 to clinical parameters of disease may provide insights into disease pathogenesis and provide important biomarkers for use in drug development and clinical trials for therapies targeting TDP-43 function.

## 5. Conclusions

In this study, we describe the development and validation of an MSD-based immunoassay for the detection of full-length TDP-43 in human biofluids, which shows high sensitivity and specificity for TDP-43 in blood samples. The assay has potential clinical utility for the diagnosis and monitoring of TDP-43-associated disorders, as well as for the development of therapeutic strategies targeting TDP-43. Our study highlights a significant decrease in full-length TDP-43 levels in ALS plasma and serum, offering a promising avenue for further research into the molecular mechanisms of ALS and FTD. Further studies are necessary to validate the clinical application of the assay in larger cohorts and to investigate its performance in longitudinal studies. Understanding the specific molecular events that lead to decreased full-length TDP-43 in ALS blood samples could provide valuable insights into disease mechanisms and potentially identify novel therapeutic targets.

## Figures and Tables

**Table 1:** An overview of the conditions optimized for the current TDP-43 assay. This table presents the optimized parameters for the Meso Scale Discovery (MSD) assay using the specified antibody pair. The parameters include antibody concentrations, incubation times, buffer compositions, and detection settings. Each parameter has been meticulously fine-tuned to ensure maximum assay sensitivity and specificity for the target analyte.

Assay Parameter	Variables Tested		Variable Selected
Antibodies	6 antibodies in all combinations (see Figure 1B)		Capture: PtG Rb 10782 Detection: R&D Ms 671818R
Blocking reagents	Casein/TBS 1% BSA in Casein/TBS Iron Horse Assay Diluent/PBS TRIPLE Block (1% Casein, 1% BSA and 1% fish gelatin) +Tween-20		1% BSA in Casein/TBS
Blocking time and temperature	2 hrs at 37°C, Shaking (750rpm) Overnight at 4°C, No Shaking 2 days in 4°C, No Shaking		2 hrs in 37°C, Shaking (750rpm)
Diluents	Casein/TBS Casein/TBS+Triton Casein/TBS+Tru Block Casein/TBS+Tru Block+Triton Casein/TBS+Tru Block+Tween-20 Iron Horse Assay Diluent/PBS		Iron Horse Assay Diluent/PBS
Sample dilutions for plasma	2-fold 4-fold 8-fold 16-fold	32-fold 64-fold 128-fold 256-fold	4-fold
Sample dilutions for serum	2-fold 4-fold 8-fold 16-fold	32-fold 64-fold 128-fold 256-fold	4-fold
Capture antibody concentrations	0.5 μg/mL 0.75 μg/mL	1.0 μg/mL 1.5 μg/mL	1.5 μg/mL
Detection antibody concentrations	0.5 μg/mL 0.75 μg/mL	1.0 μg/mL 1.5 μg/mL	0.5 μg/mL
SULFO-Tag concentrations	0.5 μg/mL 0.75 μg/mL	1.0 μg/mL 1.5 μg/mL	1.0 μg/mL

## Data Availability

The data that support the findings of this study are available via a data use agreement from the corresponding author upon reasonable request.

## Supplementary Materials

The following supporting information can be downloaded at: https://www.mdpi.com/article/10.3390/bios14120578/s1, Supplementary Figure S1: A. Scheme of antibody binding in the MSD assay as described in this protocol. B. Overview of the steps in the MSD assay. Supplementary Figure S2: The workflow for immunodepletion of endogenous TDP-43 from human biofluid samples. Human biofluid samples pooled from healthy controls were subjected to immunoprecipitation to remove endogenous TDP-43 using anti-TDP43 mouse monoclonal antibodies 671818R and 982022 or a control mouse monoclonal antibody (Ms IgG). The immunodepleted samples were analyzed using our MSD assay to quantify full-length TDP-43, and the results were compared to those from non-depleted control samples (no antibody). Supplementary Figure S3: Optimization of TDP-43 immunoassay parameters. A. The heatmap illustrates the signal-to-noise ratio of various antibody pairs tested for TDP-43 detection. Red indicates the highest signal-to-noise ratio, while yellow represents the lowest. B. Comparative analysis of six different sample diluents tested to optimize the assay conditions for TDP-43 detection. The black dashed box represents the antibody pair selected in the assay. The 20% IHAD (orange bar) provided the highest signal intensity for a 5 ng/mL TDP-43 sample and very low background for the buffer-only condition (0 ng/mL). Each bar represents an average of replicate sample wells. The black arrow represents the diluent selected in the assay. C. Various blocking buffers were tested to minimize non-specific binding. The impact of each blocking agent on signal intensity is shown, with each bar representing an average of two replicates. The black arrow represents the blocking buffer selected in the assay. D. The effects of different blocking times and temperatures on assay performance were evaluated to determine optimal conditions for reducing background noise. With a 20 ng/mL TDP-43 sample, all test conditions generated a similar signal intensity, with 2 hrs at 37C being the highest level (gold bars, using left y-axis scale), and all conditions yielded a similar background signal intensity of ~150 (blue bars and right y-axis scale). The black arrow indicates the blocking condition chosen for the assay. E, F: TDP-43 levels were assessed in both plasma (E) and serum (F) samples at varying dilutions to define dilution conditions that reduce the matrix effect. The dashed box highlights the dilution factor selected that provided reliable and consistent results across samples for each matrix. Supplementary Figure S4: Optimization of TDP-43 capture and detection antibody concentration in biofluids. A: The titration curve shows the optimization of the capture antibody concentration, ranging from 0.5 µg/mL to 1.5 µg/mL, to determine the ideal concentration for TDP-43 detection. The black arrow denotes the concentration of the capture antibody selected for the assay. B: The detection antibody was titrated from 0.5 µg/mL to 1.5 µg/mL, tested at three different concentrations of the capture antibody (0.5 µg/mL, 1.0 µg/mL, and 1.5 µg/mL) to identify the optimal pairing of capture and detection antibodies. The black arrow indicates the selected concentrations of the capture (1.5 µg/mL Rb 10782-2-AP) and detection (0.5 µg/mL 671818R) antibodies for the assay. C: Titration of the SULFO-tagged antibody was performed at two detection antibody concentrations (0.5 µg/mL and 1.0 µg/mL) to optimize the assay’s signal detection and dynamic range. The black arrow indicates the concentration of the SULFO-tag (1.0 µg/mL) selected for the assay.

## Appendix A

**Human TDP-43 Assay Protocol**

**Reagent Preparation:**

Bring all the reagents to room temperature before use. Polypropylene low-binding tubes must be used in all the steps.

1. **Coating Antibody Solution**: Make 1.5 µg/mL TDP-43 Rabbit Polyclonal antibody in PBS. *The antibody concentration may vary across the different batches, and it is good practice to perform a titration for each new batch. For example, if the antibody is 400 µg/mL, take 18.75 µL of antibody Add into 5 mL of 1X DPBS (Gibco, Catalog#14190-144). Mix well. *Prepare fresh solution before each use.
2. **Blocking Solution**: Add 2 g of BSA into 200 mL of 1x Tris Buffered Saline (TBS) with 1% Casein. Make sure that BSA is completely dissolved. Store at 4 °C up to 3 months.
3. **Sample Diluent**: Make 20% IHAD. Take 20ml of IHAD and add 80ml of 1X PBS. Store at 4 °C up to 3 months.
4. **Wash Solution**: Dilute 100 mL of 10X PBS (final concentration 1X) with 900 mL of DI Water.

Add 10 mL of 10% Tween-20 (final concentration of 0.1%). Store at RT up to one month ***Clean the wash solution bottle every 4 weeks.**

5. **Standards**: Take Standard Stock out from −80 °C and equilibrate at RT for 15 min. Add 980 µL of sample diluent to Standard Stock: S1 (Standard Stock is 1000 ng/mL, 20 µL/ vial). Gently mix by vortex using short pulses. Equilibrate the standard stock at RT for 15 min with gentle agitation prior to making dilutions.

Label 7 of 1.5 mL Protein LoBind tubes as S2 to S8 and add 600 µL of Assay Diluent per tube. Use S1 to produce a dilution series for preparing the next standards as shown in the figure below. Pipette 200 µL of S1 to S2 containing 600 µL Assay Diluent. Vortex and spin each tube before transfer. The tube S8 serves as the zero standard (0 pg/mL).
***Make Standards and QCs fresh. Do not store or reuse!**

6. **Sample Preparation**: Take 40 µL aliquot samples from −80 °C to RT and let them thaw for not more than 30 min. When thawed completely, dilute the sample four times by adding 120 µL Assay Diluent immediately. Vortex well and let them equilibrate for at least 15 min. During the 15-min equilibration time vortex every 5 min and after the last vortex, spin for 60 s.
7. **Detection Antibody Solution**: Prepare mouse mono anti-human TDP-43 at final concentration of 0.5 µg/mL in 1% Casein/TBS. Mix well by gentle vortex.

***Prepare the fresh detection antibody solution immediately before use.**

8. **Sulfo-tagged anti-mouse solution**: Make 1.0 µg/mL Sulfo-tag labeled anti-mouse in 1X TBS with 1% Casein. Mix well. ***Prepare 15 min before use.**

**TDP43 plate preparation**

1. Coating the MSD plate
  - Load the plate with 40 µL of 1.5 µg/mL coating antibody (Rabbit poly anti-human TDP-43) prepared in 1X PBS.
  - Seal the plate with microplate sealer and incubate overnight at 4 °C (no shaking).
2. Blocking the MSD plate
  - Wash the plate four times with 250 µL of 1X PBST.
  - Remove any remaining wash buffer by inverting the plate and assertively tapping it against clean paper towels.
  - Add 150 µL block buffer (1% BSA, 1% Casein TBS) to each well and seal the plate with new microplate sealer. Incubate the plate for 2 h at RT with shaking at 750 rpm using a plate shaker.
  - The coated MSD plates can be stored in Blocking Solution up to 2 weeks at 4 °C.

**Assay Procedure**

*Bring all the reagents, standards, samples and MSD plates to RT before the starting the assay

1. Wash and Add Sample
  - Wash the assay plate coated with the capture antibody containing the block buffer, four times with 250 µL of 1X PBST.
  - Load 40 µL of Standards and diluted Samples to the designated well with sample diluent in the antibody coated-MSD plate. Seal the plate with a microplate sealer and incubate at 4 °C overnight (12–20 h) on a plate shaker at 750 rpm
2. Wash and Add Detection Antibody Solution
  - Take out the plates from 4 °C and bring to RT for 30 min.
  - Wash the plate four times with 250 µL of 1X PBST.
  - Add 40 µL of Detection Antibody Solution (0.5 µg/mL) per well. Incubate at 37 °C for 2 h by shaking at 750 rpm using a plate shaker.
  - Wash the plate four times with 250 µL of 1X PBST.
  - Add 40 μL of 1 µg/mL Sulfo-tagged anti mouse antibody (1:1000) diluted in 1% Casein TBS. Seal the plate with a new adhesive film and incubate the plate at 37 °C for 60 min on a plate shaker at 750 rpm. Avoid exposure of plate to direct light.
3. Wash and Read
  - Wash the plate four times with 250 µL of 1X PBST.
  - Add 150 µL of MSD Read Buffer A to each well. Incubate for 5 min at room temperature.
  - Read plate with the MSD MESO QuickPlex SQ 120.
