# Supplementary material for: Development of a Sensitive and Reliable Meso Scale Discovery-Based Electrochemiluminescence Immunoassay to Quantify TDP-43 in Human Biofluids
Source: Biosensors (Basel). 2024 Nov 28;14(12):578. doi: 10.3390/bios14120578 (PMC11675039; doi:10.3390/bios14120578)

## Supplementary figure S1

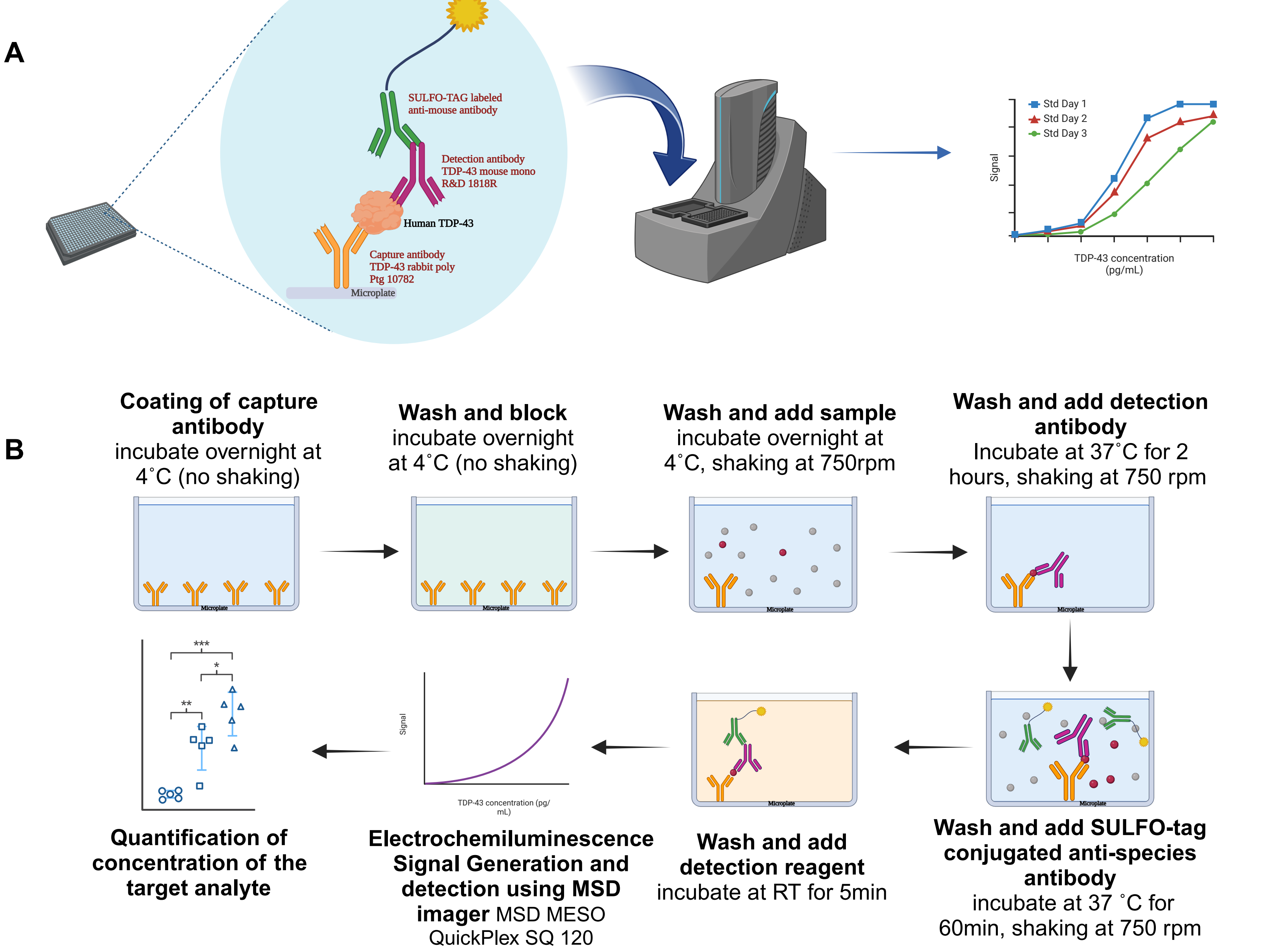

Supplementary figure S2

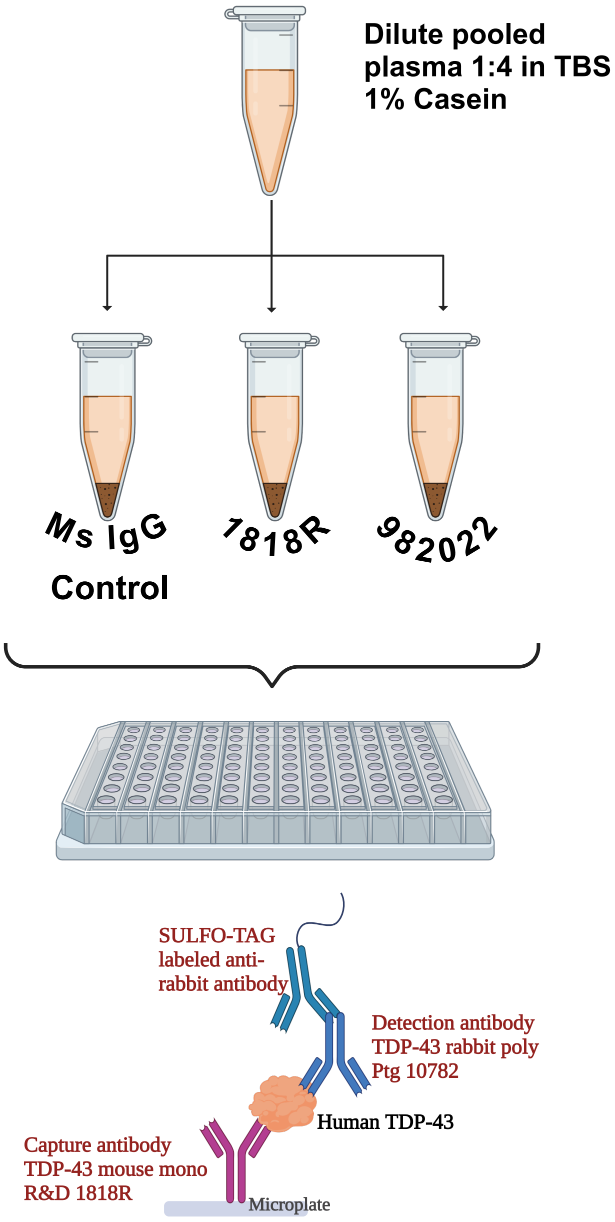


Bind Dynabeads with 4 µg of Ms TDP-43 Ab/ IgG, wash, add 200µL of 1:4 plasma, deplete overnight at 4°C with high agitation

Remove supernatant from beads, load on an MSD plate coated with Ms mono TDP-43 capture antibody (1818R)

Assess depletion efficiency with Rb poly 10782 detection antibody

# Supplementary figure S3

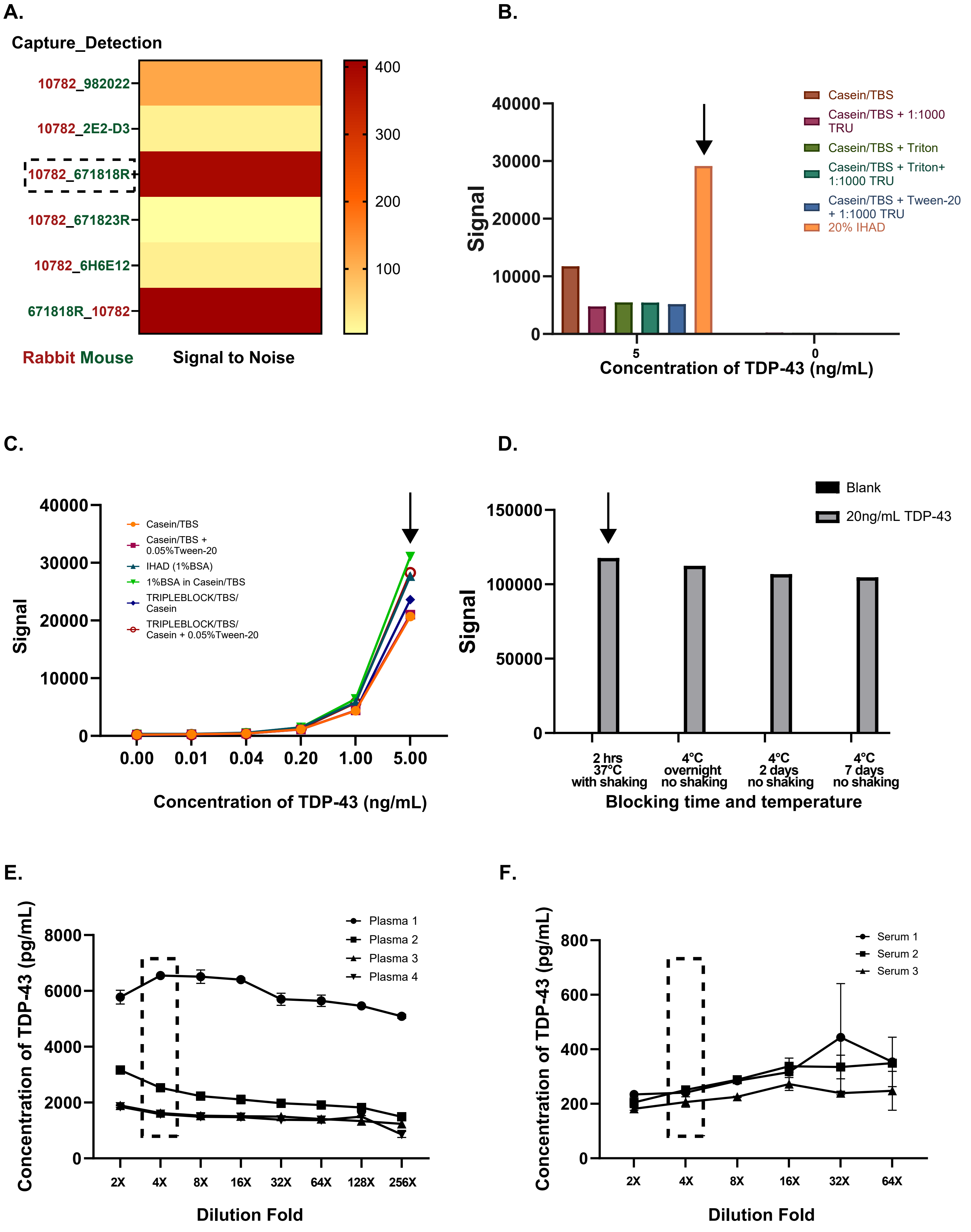

**Supplementary figure S4**

A

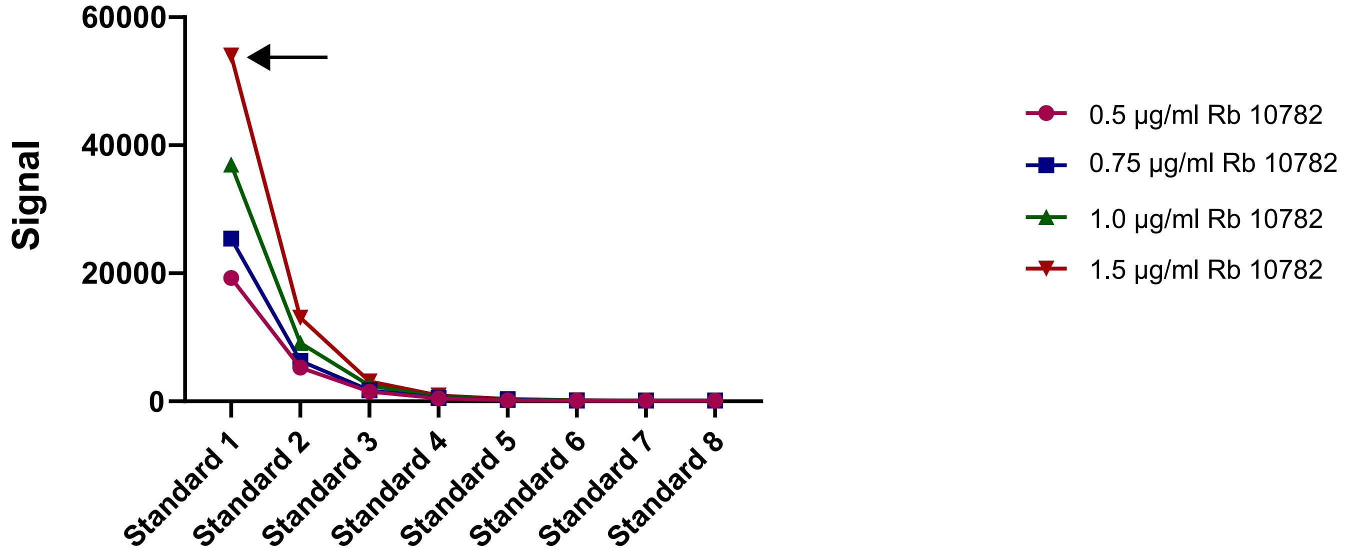


B

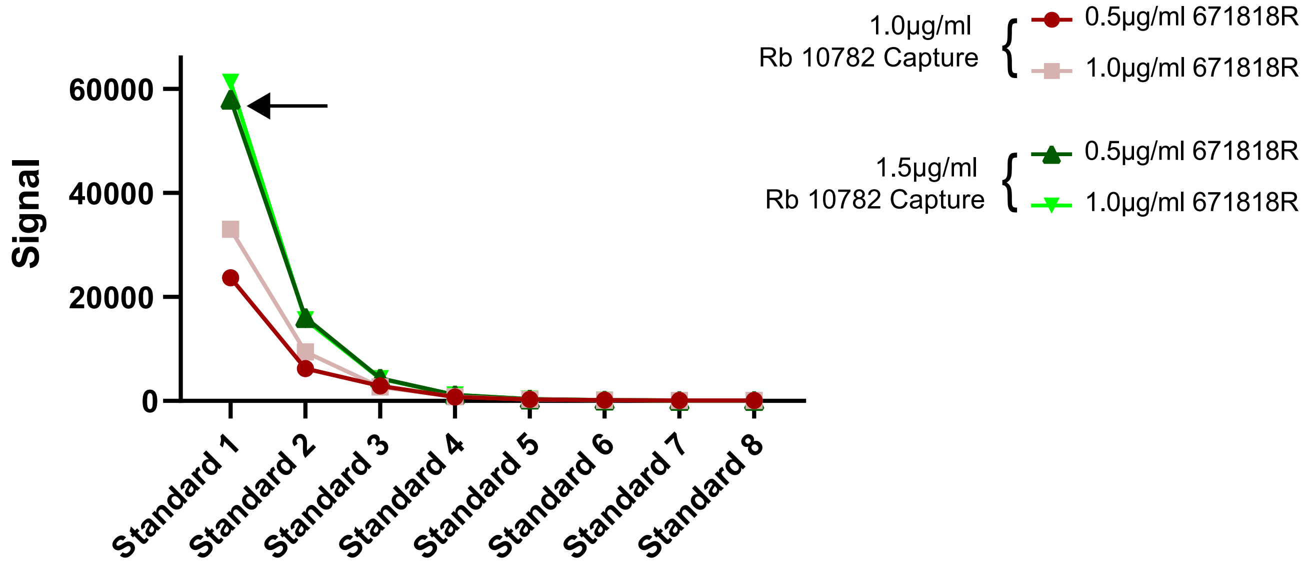


C

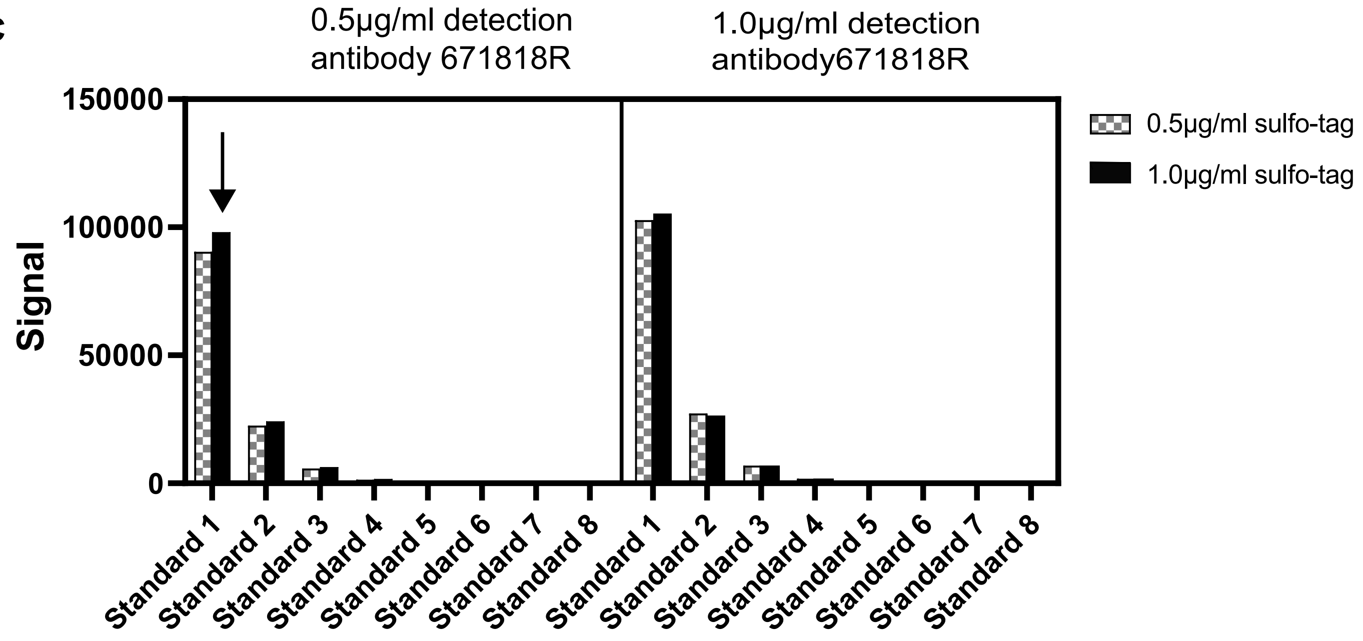

Supplement: Supplementary file 1 [file biosensors-14-00578-s001.zip › biosensors-3228005-Supplementary.pdf]
